# Online joint localization without user interactions

**DOI:** 10.1038/s41598-023-49816-5

**Published:** 2023-12-15

**Authors:** Yanfen Le, Jintian Ou, Yuanhao Chen, Heng Yao

**Affiliations:** School of Optical-Electrical and Computer Engineering, University of Shanghai for Science and Technology, Shanghai, 200093 China

**Keywords:** Engineering, Mathematics and computing

## Abstract

Wi-Fi fingerprinting has been a popular indoor localization due to the widespread layout of indoor WLAN. However, the signal fluctuations in the complex environments make it difficult to maintain high accuracy localization for the received signal strength (RSS) fingerprinting. Various positioning solutions have emerged to address this challenge, either working in stand-alone mode or in collaborative mode. In the former case, the user only utilizes his own RSS observation to request location service, while the latter usually requires information transfer between users. Considering the spatial correlation of wireless signal distribution, we propose an online joint localization scheme (JointLoc) that does not require direct interaction between users. The fact that the signals observed by users in physical proximity characterize the surroundings is used by JointLoc to identify neighboring users for joint localization. Besides this, JointLoc further integrates a novel subset-based localization scheme, thus the influence of anomalous RSS signals is eliminated before making the final location decision. We have fully evaluated the performance of JointLoc in two RSS datasets collected in real environments. Compared with conventional algorithms and the latest ones, results show that JointLoc is robust against signal fluctuations, and achieves good localization accuracy.

## Introduction

Indoor location-based services (LBS) have received increasing attention in recent years, such as smart buildings, personal intelligence services, location tracking and other IoT-based applications that require the location information of users or targets. Many technical means based on different forms of available signals, such as light, magnetic, sound, image, radio, etc., can be used to achieve localization in indoor environments^1–4^. Due to the deployability, Wi-Fi fingerprinting has become one of the most promising techniques and has been studied extensively in the last decade^5–7^.

Fingerprinting localization usually has two phases: offline phase and online phase^8^. In the offline phase, a radio map containing fingerprints, i.e., signal vectors of the received signal strength (RSS) from Wi-Fi access points (APs) at each reference point (RP), is generated with the site survey, and a certain mapping model between RSS signals and physical locations in the site can be further established by various learning or training methods. And in the online phase, at any time desired, the mobile user (client) measures the RSS signal and sends it to the server to request location information, while the server estimates the position of the user according to the mapping function and returns it to the user. The traditional localization algorithm is weighted *k*-nearest-neighbors (WKNN)^8^, which is based on the similarity between the user's signal vector and the fingerprints of the reference points to find the top *k* nearest RPs whose fingerprints closely match the user measured one. Then WKNN computes the weighted average of the locations of all the *k* RPs to localize the user.

Obviously, the localization accuracy is closely related to how well the RSS signal acquired by the user matches the fingerprints in the radio map. Various machine learning algorithms have been applied to fingerprinting in an attempt to learn a robust and accurate localization scheme from the noisy and fluctuating RSS signals collected offline^9–11^. However, when performing online signal measurements, various factors in the dynamic indoor environment make the signal distribution in the local area where the user is located differ from the offline fingerprints. For example, crowd activity causes short and sharp fluctuations in the RSS signal of the specific APs covering the active area, or, turning off several APs makes the similarity between the RSS signal vector and the fingerprints of RPs around the user decrease significantly, thus introducing large location ambiguities. To address such problems, most of the existing methods collect signals from each AP multiple times and then simply take time averaging or filtering, estimator, etc. to dynamically weaken the impact of these anomalous signals on localization^12,13^. However, it does not work for the localization that scans RSS signal only few times or even once.

In many scenarios, there are a number of mobile users requesting for localization services at the same time, and then some previous works have proposed to conduct a kind of collaborative localization to improve the performance. Besides the location information obtained by individual users themselves, the physical distance or spatial proximity in between users provides additional spatial constraints, thus enabling a more accurate definition of the location of the user. However, apart from the acquired RSS signal, they all need to build a transmission-reception link between users with additional wireless unit such as ZigBee, Bluetooth, Ultra-wideband sensor, etc.^14–16^, to determine the ranging measurement.

Motivated by the above challenges, we propose an online joint localization scheme using only RSS vectors of the requesting users, called JointLoc in this paper. Scenarios with multiple users requesting for location from the server are considered, and when one of these users is called the target user (target), the others who also present location requests in the same subarea are called co-users. The proposed algorithm is based on two practical observations. Firstly, the signals of all access points are not necessarily required to complete the localization, which can also be achieved by partial fingerprint matching; secondly, usually several of the AP signals received by the user have drastic fluctuations, and the RSS vector containing these components would cause large localization error. Therefore, the basic idea of JointLoc is to generate the corresponding sets of candidate RPs based on multiple signal subsets, and then find the location relationship of these candidate RPs, target and the co-users according to their received signal strength from certain AP, so as to redefine the nearest neighboring RPs of the target and obtain the corrected estimated positions. Those positions corrected with fluctuating AP signals may still deviate from the true position of the target, so a position density is used to reject these abnormal or deviated estimated positions.

The JointLoc has the following novel contributions:

1. Neither extra hardware for inter-user measurements nor offline clustering is required to identify neighboring users for joint localization. JointLoc determines the co-users around the target simply with the RSS signals submitted by each user when requesting location from the server; in other words, the process of joint localization does not require direct interaction between peer users and is transparent to them.
2. The algorithm fuses two novel techniques to ensure positioning accuracy. One is a density-based subset localization scheme, which divides the detected RSS vector into multiple subsets for position estimation, and locates the user with the most densely estimated positions, thereby eliminating the effect of deviated estimated positions due to anomalous signals; the other is to accurately select the nearest neighboring RPs from the candidate ones with the help of co-users in each subset localization. In this way, JointLoc can accurately locate the user even if he or she only performs single signal scan.
3. The proposed JointLoc is simple and powerful. No model training is required in the offline phase except for the site survey. Only applying WKNN localization algorithm several times with different parameters in the online phase, JointLoc obtains good localization performance thanks to the above novelty.

We conduct extensive experiments on RSS datasets collected in two real environments. Our results show that JoinLoc is simple but has good localization performance, and the parameters involved in the scheme have low dependence on the application environment and can be set based on empirical values. This is beneficial for the deployment of JointLoc in certain applications with limited resources.

## Related work

In this section, we review mostly the work related to the elimination of anomalous signal influences and collaborative localization in indoor Wi-Fi fingerprinting schemes, which have mainly focused on exploring different solutions to improve the accuracy and robustness of localization.

### Stand-alone localization with outlier mitigation

In a noisy indoor environment, the major challenge of fingerprinting-based localization is to achieve a reasonable accuracy using RSS vectors that may vary greatly due to the fluctuating characteristics of the wireless signal. Generally, signals used as fingerprints are collected over a long-time span with sufficient samples to capture the RSS fluctuations and environment disturbances. Besides, many researches are devoted to extract reliable features from offline fingerprints to build good mapping functions^17–19^. Considering the nonlinear relationship between RSS and signal propagation distance, kernel-based learning methods are introduced in such regression models to build more accurate functions^20^. The work in^21^ applies a deep neural network to tackle this problem, which is conducted with staked denoising autoencoder pre-trained to learn reliable features from widely fluctuating Wi-Fi data. Works in^22–25^ utilize neural networks with different structures, including recurrent neural networks, backpropagation neural networks and convolutional neural networks to learn the signal characteristics of specific areas in order to achieve good adaptability and accuracy.

The above methods are dedicated to the training with offline noisy fingerprints, while others also focus on mitigating noise in the online measurements^13,26,27^^.^ The related work to our study is LAAFU^27^, where a cluster-based subset localization scheme is proposed for localization with altered APs. Randomly generated RSS subsets are used to estimate the user’s location, and then the locations forming a dense cluster are identified to target the user. We learned the idea from LAAFU that the locations estimated with RSS subsets without altered APs form a dense cluster, or otherwise disperse. Based on this, we have made two important improvements to make the localization scheme simpler and more efficient. Firstly, JointLoc uses the RSS subsets to select candidate RPs for subsequent joint localizations, rather than directly for user location estimation. Secondly, JointLoc uses a simple but more efficient density-based selection method to quickly find the most confident ones from multiple estimated locations. However, all these schemes, except JointLoc, work in the stand-alone mode, i.e., the user only utilizes the signals observed by himself to accomplish the localization.

### Collaborative localization

Spatial proximity detected using some proximity sensors or ranging measuring units indicates whether two users are close to each other, which can be exploited to build a radio map of the environment and localize mobile users^14–16,28,29^. Thus, in the last two decades, collaborative localization schemes have been much studied for improving fingerprinting performances, which mainly exploit the spatial relations among multiple mobile users to apply constraints on the user’s position^14,30^. The work in^14^ is supposed to be the first to propose collaborative localization as an approach to enhance fingerprinting position estimation, where nearby neighbors are detected using ZigBee radios and the estimated position of target node with low confidence is corrected with more accurate position information from nearby neighbors. The work in^31–33^ fuses the mutual distances between mobile users with the noisy fingerprint signals to build a more accurate location model. However, in addition to acquiring the Wi-Fi RSS signals, distance measurements like sound ranging, Bluetooth ranging, or dead reckoning must also be performed either with the help of sensors built in the mobile device or additional hardware. In^34,35^, a novel online collaborative localization (OCLoc) has been proposed, in contrast to above methods, which only needs the RSS signals from nearby users and thus no additional hardware or measurement exchange between peer users is involved. We take inspiration from this idea and make improvements to enable a simpler and more effective confidence evaluation of the candidate RPs based on neighboring users, and its integration with RSS subset localization promises the accuracy of the proposed JointLoc. We validate the performance of JointLoc with our own data and a publicly available dataset both collected in real environments.

## Proposed system

### System overview

**Figure 1:**
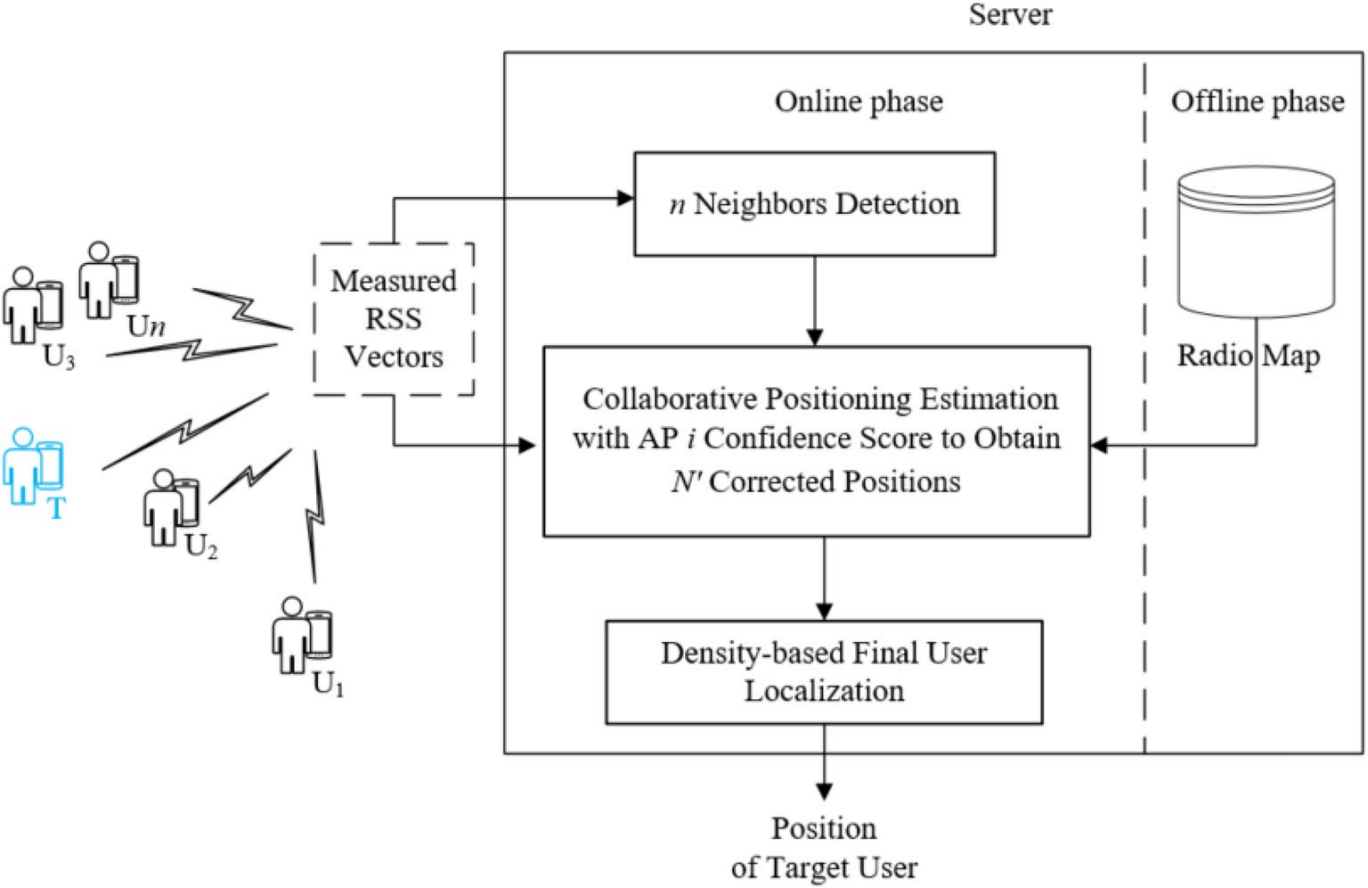
The framework of proposed JointLoc.

The proposed JointLoc scheme is dedicated to improve the localization accuracy and robustness by leveraging the signal correlation introduced by the spatial proximity of the online peer users. Figure 1 shows the framework of designed JointLoc, which consists of the following three modules in the online phase: *Neighbors Detection*, *Joint Positioning Estimation*, and *Target Localization*, in addition to the site survey and the establishment of the radio map in the offline phase.

Let *M* be the number of RPs in the survey site and *i* be the index of RPs. Denote the coordinate of RP *i* as *l*_*i*_. Then the set of RP locations is denoted as **L** = {*l*_1_, *l*_2_, …, *l*_*M*_}. The fingerprint of RP *i* is

1 $$ F_{i} = [f_{i1} ,f_{i2} ,...,f_{iN} ],1 \le i \le M, $$

where *f*_*ij*_, 1 ≤ *j* ≤ *N*, is the measured RSS value from AP *j*. Then all fingerprints in the site are denoted as **F** = {*F*_1_, *F*_2_, …, *F*_*M*_}, which are stored into the radio map together with **L**.

The online work is summarized as follows:

1. *Neighbors Detection* identifies the proximity of users who are requesting for positioning services without the need for offline clustering of fingerprints.
2. *Joint Positioning Estimation* obtains multiple corrected estimated positions, each of which corresponds to the signal of an AP detected online by the target. For each AP, possible candidate RPs are chosen according to an RSS subset and then are attached confidence scores with the help from measures from the co-users. RPs with higher confidence scores are selected and then fed to the WKNN algorithm to generate a corrected position.
3. *Target Localization* finds the dense estimated positions and their centroids are returned as the final localization result for the target.

### Neighbor detection

**Figure 2:**
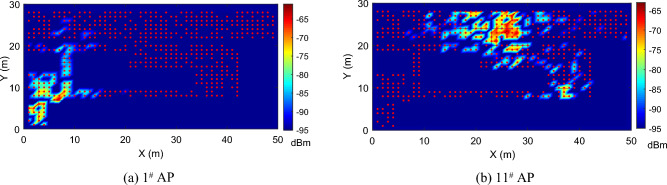
The fingerprint heat maps of two APs. Red dots are reference points.

For each target, the *Neighbor Detection* finds its neighbor requesting users within the same subarea. Instead of fingerprint clustering, we use the similarity between the coverage vectors of users’ RSS signals to identify users adjacent to the target as co-users for the following joint localization. This is based on an observation that each AP has a certain signal coverage, and users in close proximity usually detect a similar set of APs. We show the signal heat maps (spatial distribution of RSS signals in dBm) from two APs in Fig. 2. It is clear the two heat maps are markedly different. If two users detect these two APs separately, it is almost certain that they are not neighboring users. Therefore, we use the similarity of the set of detected APs to find users in the location neighborhood.

Let $C = [I_{1} ,I_{2} ,...,I_{N} ]$ denotes the coverage vector, where *I*_*j*_ = 1, 1 ≤ *j* ≤ *N*, if the user detects AP *j*, and 0 otherwise. JointLoc finds the top *n* users whose coverage vectors closely match the target’s one among all requesting users. The comparison between coverage vectors is based on the Hamming distance, i.e.,

2 $$ {\mathbf{H}}(C_{t} ,C_{u} ) = \sum\limits_{j = 1}^{N} {\left| {I_{tj} - I_{uj} } \right|} , $$

where C_*t*_ and C_*u*_ are the coverage vectors of the target and one of requesting users, respectively. Let **U** = {U_1_, U_2_, …, U_*n*_} be the selected co-users for the following joint positioning estimation with candidate RPs.

### Joint positioning estimation

Multiple corrected estimated positions are obtained in this section, which are generated with RSS subsets and corrected with co-users according to signals from each AP detected by the target. If both the RP *i* and the target receive stronger or weaker signals from AP *j* than a co-user receives, usually they are trend to be closer or further away from AP *j* than the co-user. Then we consider RP *i* credible as one of the nearest neighboring RPs of the target from this co-user’s point of view, otherwise, it is not credible. Each candidate RP is given a confidence score based on the credibility judgments of these co-users, and this determines whether it is used as a nearest neighbor (NN) to estimate the location of the target.

There is a prerequisite for using co-users to correct the estimated position, that is, the selected candidate RPs include those around the real position of the target, otherwise it would be futile for the co-users to make any effort to make the correction. JointLoc generates multiple sub-radio maps by randomly selecting subsets of detected APs, whereby several different subsets of RPs are chosen as candidates to avoid the situation where the selected candidate RPs are not near the target's real position, caused when there is a large perturbation in the RSS signal, and thus to weaken the influence of the anomalous signals on the accuracy of the estimated position.

#### Candidate RPs selection

We consider the measured RSS signals of APs detected by the target. For each AP detected, JointLoc selects a set of candidate RPs for the target localization. Specifically, a random subset sampling of APs is first conducted. Let **P** = {AP_1_, AP_2_, …, AP_*N*′_} be the set of APs detected by the target at requesting moment, then a subset **P**_s_ with *s* APs is randomly drawn from **P**. Corresponding RSS subset vector $\hat{V}_{t}$ is defined as

3 $$ \hat{V}_{t} = [v_{t1} ,v_{t2} ,...,v_{ts} ] $$

where *v*_*tj*_ is the RSS signal from AP *j* ∈ **P**_s_. We also draw a sub-radio map only containing APs in **P**_s_ from the whole radio map, i.e.,

4 $$ \hat{F}_{i} = [f_{i1} ,f_{i2} ,...,f_{is} ],i = 1,...,M $$

where *f*_*ij*_, 1 ≤ *j* ≤ *s* is the recorded RSS fingerprint of AP *j* at RP* i*. The signal distance between RSS vector $\hat{V}_{t}$ and $\hat{F}_{i}$ is computed, i.e.,

5 $$ D_{i} = \left\| {\hat{V}_{t} - \hat{F}_{i} } \right\|{ = }\sqrt {\sum\nolimits_{j = 1}^{s} {\left( {v_{tj} - f_{ij} } \right)^{2} } } ,i = 1,...,M $$

We select *k* RPs with the top-*k* smallest signal distances as the set of candidate RPs, denoted as **R** = {RP_1_, RP_2_, …RP_*k*_}. Each of the top *k* RPs is given a weight *w*_*i*_ = 1/D_*i*_ for locating the target. For WKNN algorithm, the target is located to the weighted center of these *k* RPs’ locations, i.e.,

6 $$ l = \sum\limits_{i = 1}^{k} {\frac{{w_{i} }}{W}} l_{i} ,\begin{array}{*{20}c} {} & {W{ = }\sum\limits_{i = 1}^{k} {w_{i} } } \\ \end{array} . $$

where *l*_*i*_ is the coordinate of the selected candidate RP *i*. Unlike it, JointLoc utilizes the co-users to judge the credibility of each candidate RP and finds the most credible ones from them for target localization.

#### Joint localization with confidence score

Given above selected candidate RPs, JointLoc makes a joint determination of credibility of each RP with the observation of each co-user. The basic idea of this part is based on the fact of signal attenuation with propagation distance. When the location of the AP is fixed, usually the farther the wireless signal travels, the weaker the signal strength. Therefore, we can infer the relative distance of two locations from the AP based on the RSS signal.

**Figure 3:**
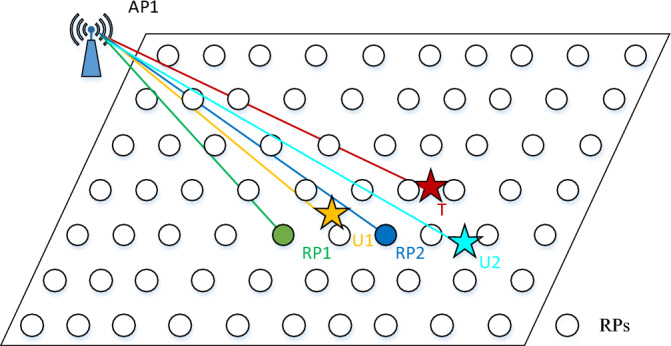
Illustration of how to compute the confidence score.

For easy of reading, we illustrate a case where the target T has only two co-users, U_1_ and U_2_, to evaluate the credibility of two members of **R**, RP_1_ and RP_2_. As shown in Fig. 3, the RSS value received at RP_1_ should be stronger than that received by U_1_, because RP_1_ is closer to the AP_1_ compared to U_1_.Similarly, RSS value received by T is weaker than that received by U_1_, that is

7 $$ f_{1} ({\text{RP}}_{1} ) > v_{1} ({\text{U}}_{1} ) > v_{1} ({\text{T}}), $$

where *f*_1_(RP_1_), *v*_1_(U_1_), and* v*_1_(T) are the RSS values of AP_1_ received at RP_1_, U_1_ and T, respectively. Thus, we infer that RP_1_ is further away from T than U_1_, and it is not credible to treat RP_1_ as the nearest neighbor of T from the viewpoint of U_1_. So, we define a negative score like

8 $$ \theta_{1} ({\text{RP}}_{1} {\text{,U}}_{1} ) = - 1. $$

The similar analysis is done to RP_2_. We get

9 $$ v_{1} ({\text{U}}_{1} ) > f_{1} ({\text{RP}}_{2} ) > v_{1} ({\text{T}}), $$

which means RP_2_ should be closer to T than U_1_. From the viewpoint of U1, it is credible to treat RP1 as the nearest neighbor of T. Thus, we define an affirmative score like

10 $$ \theta_{1} ({\text{RP}}_{2} {\text{,U}}_{1} ) = 1. $$

Similarly, we get $\theta_{1} ({\text{RP}}_{1} {\text{,U}}_{2} ) = 1$ and $\theta_{1} ({\text{RP}}_{2} {\text{,U}}_{2} ) = 1$ from the viewpoint of U_2_. If the scores from these two co-users for a particular RP are fused and sorted, we get the confidence scores *S* like

11 $$ \left\{ {\begin{array}{*{20}c} {S_{1} ({\text{RP}}_{1} ) = \theta_{1} ({\text{RP}}_{1} {\text{,U}}_{1} ) + \theta_{1} ({\text{RP}}_{1} {\text{,U}}_{2} ) = 0} \\ {S_{1} ({\text{RP}}_{2} ) = \theta_{1} ({\text{RP}}_{2} {\text{,U}}_{1} ) + \theta_{1} ({\text{RP}}_{2} {\text{,U}}_{2} ) = 2} \\ \end{array} } \right., $$

which implies RP_2_ is more credible to be selected as NN with the joint assessment of AP_1_ signal from the two co-users.

Generally, a confidence score based on AP *j* for RP *i*, 1 ≤ *i* ≤ *k* in **R** is defined as:

12 $$ S_{j} ({\text{RP}}_{i} ) = \sum\limits_{x = 1}^{n} {\theta_{j} ({\text{RP}}_{i} ,{\text{U}}_{x} )} ,j = 1,...,s. $$

where each entry of $\theta_{j} ({\text{RP}}_{i} ,{\text{U}}_{x} )$ corresponding to the credibility of RP *i* from the viewpoint of co-user U_*x*_. A higher value of *S*_*j*_ (RP_*i*_) implies that RP *i* is more credible as the NN of the target after a joint evaluation of signal AP *j* by *n* co-users. Then the top *k*′ RPs in **R** with the highest confidence scores are chosen, and fed to the WKNN algorithm with the corresponding weights. An estimated location of the target $l^{\prime}_{j}$ is returned by

13 $$ l^{\prime}_{j} = \sum\limits_{i = 1}^{{k^{\prime}}} {\frac{{w_{i} }}{W}} l_{i} ,\begin{array}{*{20}c} {} & {W{ = }\sum\limits_{i = 1}^{{k^{\prime}}} {w_{i} } } \\ \end{array} , $$

where *l*_*i*_ is the coordinate of the RP *i* of the selected *k*′ RPs.

For each AP in **P**, such process containing *Candidate RPs Selection* and *Joint Localization with confidence score* is performed once, so that we end up with *N*′ corrected positions that have been jointly estimated by co-users. The pseudocodes of the proposed *Joint Positioning Estimation* are presented in Algorithm 1.

**Algorithm 1:**
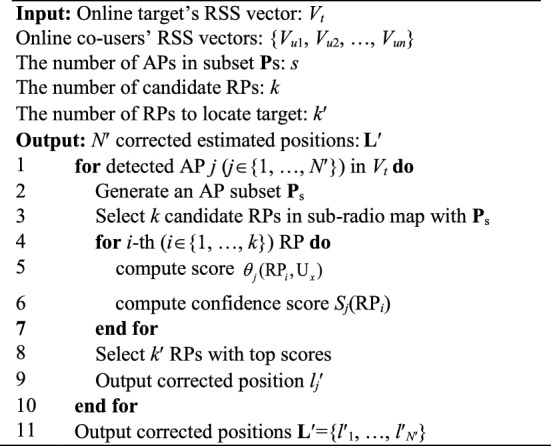
Proposed Joint Positioning Estimation

### Target localization

In principle, these *N*′ corrected positions are clustered around the real position of the target. However, fluctuation in some RSS signals and environment disturbances cause the corresponding estimated positions scattered, even with the joint localization of the co-users. JointLoc identifies the most credible ones and outputs the final localization by a local density-based clustering algorithm (DCA)^36^.

The clustering algorithm is based on the assumption that the credible estimated positions are around the real position of the target and that they are surrounded by neighbors, thus have relatively higher local densities. Specifically, for each corrected position, DCA finds its neighbors in a certain distance threshold. We define local density *ρ*_*i*_ of each corrected position $l^{\prime}_{i}$ in **L**′ as

14 $$ \rho_{i} = \sum\limits_{\forall j \ne i} {\chi \left( {d_{ij} - d_{c} } \right)} ,i = 1,...,N^{\prime},j = 1,...,N^{\prime}, $$

where $\chi \left( x \right) = 1$ if *x* < 0, and $\chi \left( x \right) = 0$ otherwise, *d*_*ij*_ is the Euclidean distance between position $l^{\prime}_{i}$ and $l^{\prime}_{j}$, and *d*_*c*_ is the preset distance threshold, which can be set according to the grid size of the RPs. Usually, *d*_*c*_ can be set to several times the grid size of the RPs. We rank all the *N*′ corrected positions according to the descending order of their local densities, then the average of the top-*n*_*f*_ corrected positions is determined as the final target location l^*^, i.e.,

15 $$ l^{*} = \frac{1}{{n_{f} }}\sum\limits_{i = 1}^{{n_{f} }} {l^{\prime}_{i} } . $$

## Experimental evaluation

We present as follows the experimental evaluations of the proposed scheme on our own dataset and the publicly available dataset^37^, both were collected in real indoor environments. The environment setups are introduced, and the results are analyzed.

### Experimental settings

**Figure 4:**
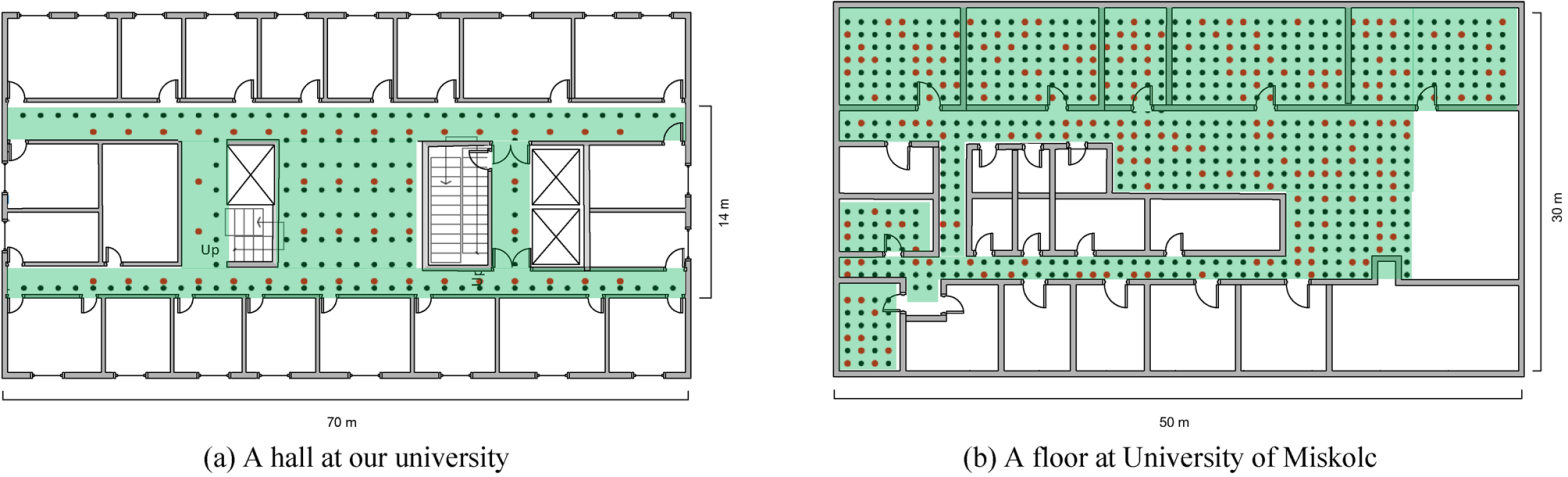
Floor maps of experimental environments.

Considering the extensive layout of the Wi-Fi networks in the buildings at present, we did not install additional APs during site survey, but used the existing APs in the buildings, and had no knowledge of these APs’ locations. The indoor layouts of the fields in our university and University of Miskolc (UM) are shown in Fig. 4, respectively. The dots in the maps indicate the locations where RSS measurements were performed. The black and red dots indicate the locations of the offline RPs and the online users, respectively.

On the map of our university (about 980 m2), we collected RSS signals on 136 RPs in 1.8 m grid size with iPhone7plus smart phone. At each RP, among the ten scanned signals, the RSS signals of each AP were averaged and recorded as fingerprints in the radio map. A total of 452 APs were detected in the whole map. For testing purpose, we also collected signals once at each of the 44 locations different from RPs as online users.

For the dataset collected in University of Miskolc, the Samsung Galaxy smart phone was used to conduct the measurements and the coverage of the map was about 1175 m2 with 1 m grid size, and there were 32 APs detected covering the floor. From the 711 sampling locations, as the dots shown in the Fig. 4b, we randomly selected 200 samples as online users and the rest as the offline RPs to build the radio map. Due to the different wall partitions and layouts of APs, different numbers of APs were detected at each RP. At our university, the number of APs detected at each RP is between 23 and 80, while at University of Miskolc, the numbers are between 2 and 17.

It should be noted that the RSS signals at each test locations are not collected at the same time. However, the neighboring test locations are sampled in a short period of time, say within half an hour. Therefore, in order to evaluate the performance of proposed scheme, we take each test location as the target user, and find the neighboring users from the remaining test locations as the co-users according to the method proposed by JointLoc.

We compare JointLoc with the following fingerprinting-based localization algorithms:

1. WKNN^8^: which is the most classic fingerprinting-based localization scheme. The weighted average of the locations of top *k* nearest RPs with the least distance in signal space is returned as the position estimation.
2. OCLoc-W^34^: which is an online collaborative localization method to calibrate the weights of the nearest neighboring RPs according to the RSS signals detected by the neighboring users. It integrates, for each nearest neighboring RP, the calibration of all requesting users and its own fingerprint distances to select the most weighted RPs to locate the user.
3. MOCLoc-W^35^: which is an improved version of OCLoc-W and is one of the latest state-of-the-art co-localization algorithms. The similarity between online users’ fingerprints is computed through the multidimensional scaling technique, and based on this, the credibility of candidate RPs is further adjusted by different online co-users.
4. 2D-GPR^38^: which is one of the state-of-the-art approaches based on Gaussian process regression. The pattern recognition algorithm introduces a joint coordinate optimization within the offline phase to obtain the desired positioning accuracy.

### Experimental results at our university

**Figure 5:**
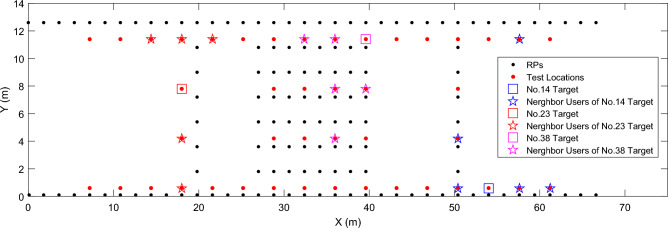
Examples of detected co-users of three targets at our university.

We first evaluatethe *Neighbor Detection*. Figure 5 illustrates examples of *n* = 5 co-users detected according to the coverage vector of the RSS signal for three targets, respectively. It is clear that, in most cases, co-users of the target can be found correctly based on the coverage vector.

**Figure 6:**
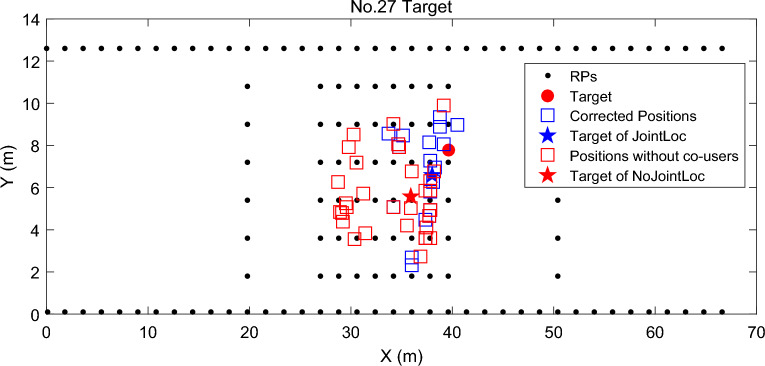
Example of localization results with and without the step of Joint Localization with Confidence Score.

We then evaluate the proposed *Joint Localization with Confidence Score* to select the real nearest neighboring RPs. A scheme denoted as NoJointLoc is introduced, which has the same modules as JointLoc but without the step of *Joint Localization with Confidence Score*. NoJointLoc directly uses the candidate RPs to get the estimated positions without the credibility evaluation from the co-users*.* Figure 6 shows the estimated positions of a given target, where *k* = 4 neighboring RPs are founded and fed to the WKNN to get the estimated positions for NoJointLoc. While for JointLoc, *k* = 10 neighboring RPs are first chosen as candidate RPs, then *k*′ = 4 RPs are selected among them after the joint localization of co-users and then get the corrected positions. These estimated positions are indicated by red and blue boxes in the figure, respectively. It is obvious that NoJointLoc yields a more dispersed estimated positions than JointLoc does, suggesting that the nearest neighbors selected with the confidence score are closer to the user's real position. By *Joint Localization with Confidence Score,* JointLoc achieves at least 14 percent localization error reduction compared with NoJointLoc.

**Figure 7:**
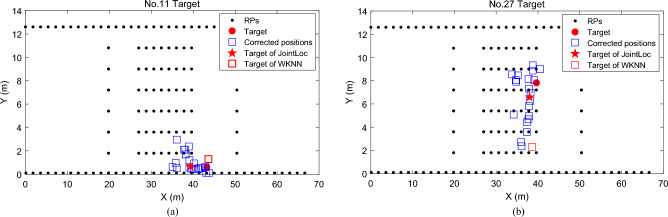
Examples of joint localization results of two targets.

Finally, we evaluate the performance of *Target Localization*. We illustrate in Fig. 7 the joint corrected positions and the final location of each target with *n* = 5 co-users and distance threshold *d*_*c*_ = 4 m for two localization requests. These joint corrected positions and the one obtained with WKNN algorithm in Fig. 7a are mostly around the real location of the target, indicating that the target acquires a little fluctuating signal and easily finds the matching RPs in the radio map. Whereas in Fig. 7b, the location with WKNN deviates severely from the target’s real location, indicating that the RSS vector collected by the target fluctuates widely and is not very consistent with the fingerprints of the surrounding RPs. We can see some of the corrected positions are still scattered even with the joint localization of co-users. However, the proposed DCA algorithm successfully locates the target near its real position.

**Figure 8:**
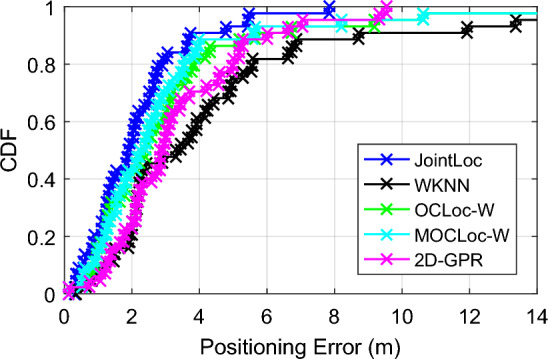
Cumulative probability of positioning errors at our university.

Figure 8 shows localization error CDFs of five different schemes. The parameter settings are as follows. In JointLoc, we empirically select *n* = 5 co-users, *k* = 10 candidate RPs, *k*′ = 4 top corrected positions and distance threshold *d*_*c*_ = 3 m. The number of nearest neighbors for WKNN is 5. While in OCLoc-W the numbers of candidate RPs and collaborative users are 9 and 3, respectively. The corresponding two parameters are set to 9 and 5 in MOCLoc-W. As can be seen, our proposed JointLoc outperforms WKNN, OCLoc-W, MOCLoc-W and 2D-GPR. We can see both WKNN and 2D-GPR achieve higher errors. WKNN only compares the signal distance between the user’s own measured signal and the offline fingerprints, which cannot eliminate the influence of signal fluctuation on localization. While for 2D-GPR algorithm, the small number of offline fingerprints and the signal perturbation may make it fail to build an accurate regression model for localization in this experimental scenario.

**Figure 9:**
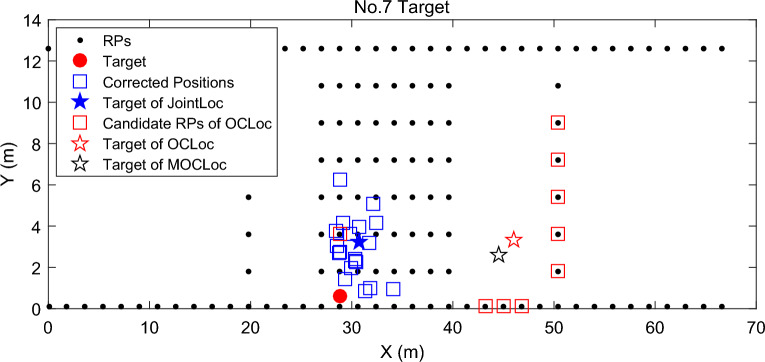
Example of specific positioning details for No. 7 target at our university.

Both OCLoc-W and MOCLoc-M select *K* candidate RPs with the top-*K* small signal distance and then adjust their weights by combining the observations of the co-users. Note that for each signal vector acquired by the target for localization query, these candidates RPs selected by OCLoc-W and MOCLoc-M are determined. The subsequent calibration with co-users is only to re-rank and re-weight these RPs. Consider the case where there are large fluctuations in the online signal, the matched candidate RPs tend to be deviated from the target’s real position. Thus, even with many co-users’ calibrations, the desired accurate estimated position cannot be obtained based on these mismatched candidate RPs. An example is given in Fig. 9, where shows *K* = 9 candidate RPs of the No. 7 target determined based on fingerprint similarity. It can be observed that the matched candidate RPs are all far away from the real position of the target, which may be due to the presence of large sudden perturbations in the online signal of this target. In this case, no matter how the subsequent calibration is performed, it is impossible for OCLoc-W and MOCLoc-W to obtain an accurate estimation based on these scattered 9 candidate RPs. Specifically, OCLoc-W and MOCLoc-W suffer from location errors of up to 17.4 m and 15.8 m, respectively for this target. Compared with them, JointLoc not only utilizes the measured signals from neighboring co-users to calibrate the candidates, but also employs a new method as described in Algorithm 1 to select the candidate RPs, which makes it possible to find the real nearest neighbors even in the presence of anomalous AP signals, while the integration with the DCA algorithm is beneficial in further eliminating the influence of anomalous signals on localization, which makes JoinLoc achieve better localization accuracy.

Further analysis shows that the average localization error of the proposed JointLoc is reduced by about 50.1%, 32.6%, 28.1% and 35.8% respectively, compared to WKNN, OCLoc-W, MOCLoc-W and 2D-GPR.

**Figure 10:**
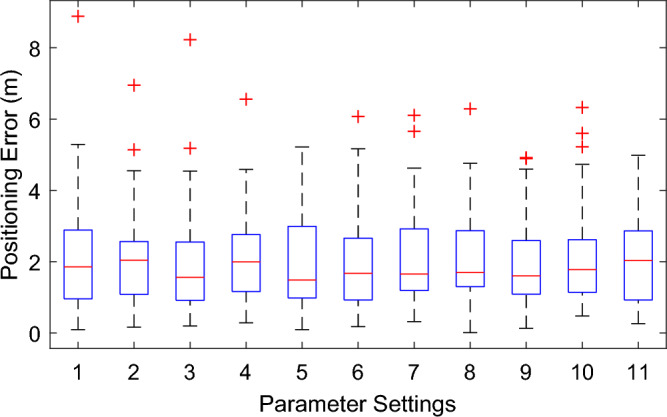
Positioning errors with different parameter settings at our university*.*

Then we perform a more intensive evaluation of the performance of JointLoc. As mentioned above, the proposed scheme involves several parameters, including the number of co-user *n*, the number of APs *s* in subset **P**_s_, the number of candidate RPs *k* for each localization, the distance threshold *d*_*c*_ and the number of estimated positions *n*_*f*_ for the final target localization. The specific parameter settings are shown in Table 1, and the corresponding positioning errors, including maximum, minimum and media values, are displayed in Fig. 8. Settings {1 to 4} are the positioning errors versus the number of co-users *n*. For this dataset, an optimal number of *n* is observed to be 6, but we can also note that setting *n* to 2 or 8 does not have a significant effect on the localization accuracy. Similar results can be observed when the number of candidate RPs *k* varies between 5 and 20, as shown in settings {3, 5 to 7}. Settings {8 to 11} cover different combinations of parameter settings, while the average positioning error is basically around 2.1 m. And results in Fig. 10 show that selecting three to four corrected positions with the highest local densities can well accomplish the localization of the target. Thus, it can be said that the proposed JointLoc overcomes the sensitivity to the parameter settings that may result from the absence of offline training. The performance of the proposed scheme can be achieved by setting the parameters based on empirical values.

**Table 1:** Values of parameters corresponding to each setting.

Settings	1	2	3	4	5	6	7	8	9	10	11
*n*	2	4	6	8	6	6	6	2	2	6	6
*k*	10	10	10	10	5	15	20	5	5	5	10
*s*	10	10	10	10	10	10	10	10	8	8	10
*d*_*c*_	4	4	4	4	4	4	4	2	3	4	3
*n*_*f*_	4	4	4	4	4	4	4	3	3	4	3

It should be mentioned that *s* must be smaller than *N*′ (the number of APs the target detects) to sure the valid generation of the RSS subset vectors. It can dynamically adjust *s* according to the *N*′, say *s* = *N*′–3, or a preset value smaller than the minimum number of APs sensed by RPs in the radio map, as adopted in the paper.

### Illustrative results in UM

We have conducted similar studies within UM over JointLoc, WKNN, OCLoc-W, MOCLoc-W and 2D-GPR. The average positioning errors of the five algorithms are 3.0 m, 3.8 m, 4.1 m, 4.0 m, and 3.7 m, respectively. In this scenario, both OCLoc-W and MOCLoc-W degrade in localization accuracy, which may be blamed on the presence of large perturbations in the online RSS signal making some of the candidate RPs to be mismatched. Thus, errors happen. 2D-GPR, on the other hand, obtains a higher accuracy, probably thanks to the fact that the large amount of the fingerprints in the offline phase allows the regression model to accurately reflect the relationship between the RSS signal and the physical location.

**Figure 11:**
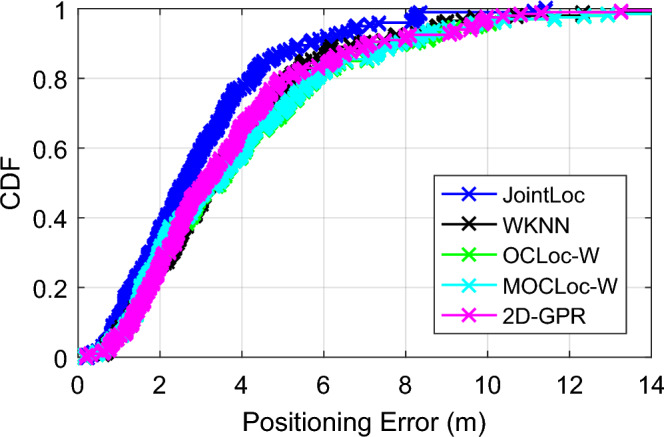
Cumulative probability of positioning errors in UM.

**Figure 12:**
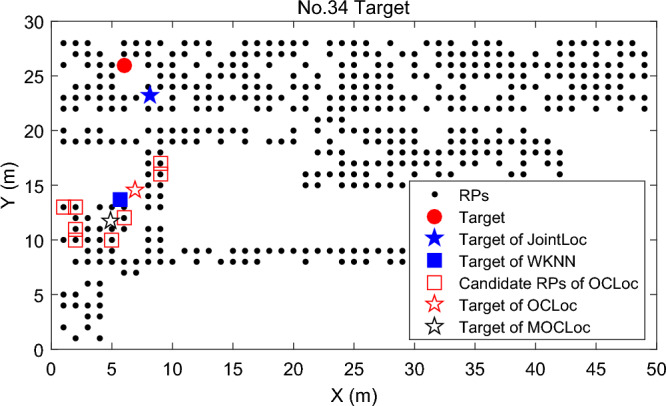
Example of specific positioning details for No. 34 target in UM.

Figure 11 shows the localization error CDFs of the algorithms, and Fig. 12 presents a more specific view of the location detail of No. 34 target. It is clear that there is a large perturbation in the online signal that makes the selected candidate RPs far away from the true position of the target. Therefore, subsequent re-ranking of these RPs in both OCLoc-W and MOCLoc-W failed to reduce the localization error. This is consistent with the results shown in the CDF figures in^34,35^, and the proposed collaborative localization in^34,35^ does not eliminate large localization errors. Experimental results show that WKNN, OCLoc-W, MOCLoc-W and 2D-GPR suffer from maximum location errors of almost 17.9 m, 24.6 m, 21.9 m and 14.7 m, respectively, while this metric is much smaller for JointLoc. In such a dynamic environment, the proposed JointLoc achieves a significant performance improvement compared to the other four algorithms.

Note that when the number of APs detected by the target at a certain location query is small, e.g., less than 5, the members of the constructed AP subset are almost the same and the corresponding RSS subset vectors do not differ much. Then JointLoc estimates locations based on a traditional fingerprinting algorithm, such as WKNN. Further analysis of the online RSS vectors shows that in three out of 200 online location queries, the users detect only 2 APs, while in another five queries, the online RSS vectors contain only three APs. There are two other queries with only 4 AP signals. For these users, JointLoc simply applies WKNN to estimate the position of the target.

**Figure 13:**
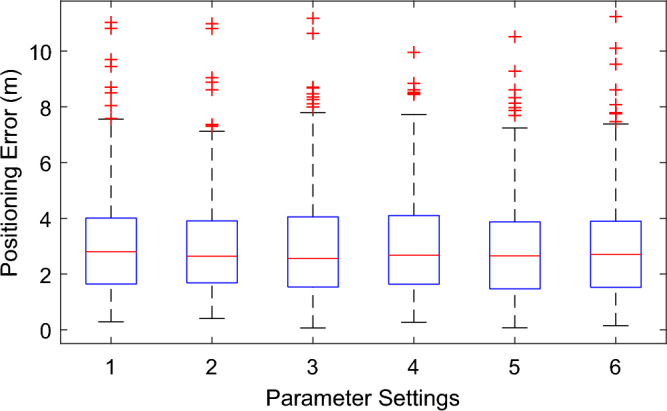
Positioning errors with different parameter settings in UM.

Figure 13 shows the localization results with different parameter settings as listed in Table 2 for the target in UM. Similarly, it is observed that there is no apparent change in the positioning results, including the maximum and median values of the errors, while the parameters of algorithm are set differently. Setting parameters based on the empirical values can guarantee a stable and accuracy localization performance.

**Table 2:** Values of parameters corresponding to each setting.

Settings	1	2	3	4	5	6
*n*	2	5	5	2	2	2
*k*	5	10	10	5	6	15
*s*	5	5	5	4	4	5
*d*_*c*_	4	4	3	4	3	3
*n*_*f*_	4	4	5	3	4	3

## Conclusion

For the scenario where there are multiple location queries from different users occurring simultaneously, in this paper, we propose an online joint localization scheme with no interaction between users requesting for positioning services, which achieves accurate indoor localization and is easy to apply due to its insensitivity to parameter settings. The RSS observations from neighboring users are used to jointly determine the credibility of selected nearest neighboring RPs, thus correcting the individual estimated position. In addition, JointLoc introduces a density-based subset localization scheme to further reduce the impact of anomalous RSS signals on localization. We have conducted experiments on field measurements from our own collected data and public dataset. The results have shown the robustness and stability of the performance of JointLoc besides the high accuracy.

Several exciting challenges arise from our current work. Firstly, how to maintain the localization system and update the fingerprint database in dynamic environments are practical issues that must be considered. We are currently investigating the use of the dispersion of a target's estimated locations to determine if the spatial distribution of wireless signals from multiple APs has changed, and from there to decide whether the site survey is needed. Secondly, long-term field studies need to be conducted to verify the localization performance of the proposed scheme in the practical indoor environment with unexpected changes. Lastly, how to address the issue of privacy preserving of user’s localization query and estimated position while maintaining the localization accuracy is challenging and interesting. Although privacy-preserving Location-Based Services have been addressed in the literature, there need more attention to the problem of privacy of indoor localization. One of our future works is to integrate the idea of improving the localization accuracy in the proposed JointLoc with the privacy preserving of the target.

## Data Availability

The datasets generated and/or analysed during the current study are available from the corresponding author on reasonable request.
